# Cross-cultural adaptation, reliability and construct validity of the Arabic Scleroderma Assessment Questionnaire in Egyptian patients with systemic sclerosis

**DOI:** 10.1007/s10067-026-08055-5

**Published:** 2026-04-09

**Authors:** Omima Ahmed El-Farra, Manal Hassanien, Amira M. Ibrahim, Wedad Mahmoud Ghazy, Enas Abolkheir Abdelaleem, Nesrin Ahmed Aboeladl, Nevine Mohannad, Khaled A. A. Abdelgalil, Gehad Gamal Maghraby, Asmaa Khalifa Ahmed, Hanan Elsaadany, Salma A. Khalil, Samah Ismail Nasef, Samar Tharwat, Osman Hammam, Shaimaa Moustafa Hafez, Nevin Hammam, Tamer A. Gheita

**Affiliations:** 1 Rheumatology, Rehabilitation and Physical Medicine Department, Faculty of Medicine, Minia University, Minia, Egypt; 2 Rheumatology, Rehabilitation and Physical Medicine, Faculty of Medicine, Assiut University, Assiut, Egypt; 3 Rheumatology, Rehabilitation and Physical Medicine Department, Faculty of Medicine, Kafrelsheikh University, Kafr El Sheikh, Egypt; 4 Rheumatology, Rehabilitation and Physical Medicine Department, Faculty of Medicine, Zagazig University, Zagazig, Egypt; 5 Rheumatology, Rehabilitation and Physical Medicine Department, Faculty of Medicine, Beni-Suef University, Beni-Suef, Egypt; 6 Rheumatology, Rehabilitation and Physical Medicine, Faculty of Medicine, Helwan University, Cairo, Egypt; 7 Internal Medicine and Rheumatology, Faculty of Medicine, Alexandria University Hospitals, Alexandria University, Alexandria, Egypt; 8 Rheumatology and Rehabilitation Department, Faculty of Medicine, Aswan University, Aswan, Egypt; 9 Rheumatology and Clinical Immunology Unit, Internal Medicine Department, Faculty of Medicine, Cairo University, Cairo, Egypt; 10 Rheumatology, Rehabilitation and Physical Medicine Department, Faculty of Medicine, Sohag University, Sohag, Egypt; 11 Rheumatology, Rehabilitation and Physical Medicine Department, Faculty of Medicine, Tanta University, Tanta, Egypt; 12 Internal Medicine Department, Rheumatology Division, Faculty of Medicine, Ain Shams University, Cairo, Egypt; 13 Rheumatology Department, Faculty of Medicine, Suez Canal University, Ismailia, Egypt; 14 Rheumatology and Immunology Unit, Internal Medicine Department, Faculty of Medicine, Mansoura University, Mansoura, Egypt; 15 Rheumatology and Rehabilitation Department, Faculty of Medicine, New Valley University, New Valley, Egypt; 16 Public Health and Preventive Medicine Department, Faculty of Medicine, Minia University, Minia, Egypt; 17 Rheumatology Department, Faculty of Medicine, Cairo University, Giza, Egypt

**Keywords:** Arabic language, Cross-cultural adaptation, Patient-reported outcome measures, Reliability and validity, Scleroderma assessment questionnaire, Systemic sclerosis

## Abstract

**Background:**

Systemic sclerosis (SSc) is a multisystem autoimmune disease characterized by fibrosis, vasculopathy, and internal organ involvement, resulting in substantial symptom burden and impaired quality of life. Reliable patient-reported outcome measures adapted to Arabic-speaking populations are lacking.

**Objective:**

To develop a cross-cultural adaptation of the English version of the Scleroderma Assessment Questionnaire to the Arabic language (Ar-SAQ) and to assess its reliability and construct validity in SSc patients.

**Patients and methods:**

Translation and cultural adaptation process followed internationally accepted guidelines for patient-reported outcomes. After expert evaluation and pretesting for semantic clarity, the final version was administered to 193 SSc patients. Test–retest reliability was assessed over four weeks using intraclass correlation coefficients (ICC). Construct validity was analysed through correlation between total and domain scores of the Arabic questionnaire and the European Scleroderma Trial and Research Group activity index.

**Results:**

Patients included 171 females (88.6%) with a mean age of 44.6 ± 10.6 years. The Ar-SAQ demonstrated satisfactory reproducibility, with ICC results ranging from moderate to excellent (0.50–0.95). The musculoskeletal, respiratory, gastrointestinal, and total index of disease status indices showed good to excellent reliability (ICC = 0.90–0.95). The vascular index showed moderate reproducibility (0.50), consistent with naturally fluctuating vascular symptoms in SSc. Construct validity was confirmed by a significant positive correlation with the European Scleroderma Trial and Research Group activity index.

**Conclusion:**

The Ar-SAQ is a reliable and valid instrument for assessing patient-reported disease status in Egyptian SSc patients and may serve as a foundation for further validation in other Arabic-speaking populations.

**Table:** 

**Key Points**
• *A culturally adapted Arabic version of the Scleroderma Assessment Questionnaire was developed following standardized cross-cultural adaptation guidelines.* • *The Arabic Scleroderma Assessment Questionnaire demonstrated good to excellent reliability and acceptable construct validity in Egyptian patients with systemic sclerosis.* • *This instrument provides a feasible patient-reported measure of disease status that may support clinical assessment and research in Arabic-speaking populations.*

**Supplementary Information:**

The online version contains supplementary material available at 10.1007/s10067-026-08055-5.

## Introduction

Systemic sclerosis (SSc) is a chronic connective tissue disease characterized by immune dysregulation, vasculopathy, and progressive fibrosis of the skin and internal organs, resulting in heterogeneous clinical manifestations and substantial morbidity and mortality [1]. Despite advances in understanding disease mechanisms and therapeutic strategies, SSc remains a major clinical challenge due to its variable presentation, unpredictable course, and lack of sensitive outcome measures [2].

In Egypt, epidemiological and clinical data on SSc remain limited. However, recent multicenter evidence suggests a relatively severe disease phenotype, with high rates of pulmonary involvement, interstitial lung disease, and pulmonary arterial hypertension, as well as frequent microvascular abnormalities on nailfold capillaroscopy [3]. These findings highlight the need for robust tools to capture the full burden of disease in this population, including patient-reported manifestations that may not be fully reflected by clinician-based indices.

The natural history of SSc is heterogeneous and is often conceptualized according to the extent of skin involvement (limited versus diffuse cutaneous disease) and disease stage. Early disease is typically characterized by inflammatory features, while later stages are dominated by vascular and structural damage [4]. However, the distinction between disease activity, severity, and irreversible damage remains challenging in clinical practice, particularly in the absence of validated biomarkers [5]. This complexity contributes to variability in assessment and management strategies among rheumatologists [6].

Several instruments have been developed to quantify disease activity, severity, or damage in SSc, including the European Scleroderma Trials and Research Group activity index (EUSTAR-AI) [7, 8] as well as the European Scleroderma Study Group Activity Index (EScSG-AI) [9]; Medsger Severity Scale [10, 11] and disease damage by SCTC-DI (Scleroderma Clinical Trials Consortium Damage Index) [12, 13]. The modified Rodnan skin score (mRSS) is widely used to assess skin induration [14]. None of them fully capture the patient’s subjective symptom burden or functional impact, and no universally accepted gold standard for overall disease activity exists [15–17]. Persistent disease activity and damage have been associated with increased mortality, underscoring the importance of comprehensive assessment approaches [13].

In this context, patient-reported outcome measures (PROMs) have gained increasing importance, as patient-reported symptoms may reflect changes in disease status over time and complement objective clinical assessments [18]. PROMs are particularly valuable in multisystem diseases such as SSc, where symptoms such as pain, fatigue, dyspnea, and gastrointestinal dysfunction substantially affect daily functioning and quality of life [19, 20].

The Scleroderma Assessment Questionnaire (SAQ) is a disease-specific PROM originally developed to assess symptom burden and functional impairment across multiple organ systems in patients with SSc [20, 21]**.** It has demonstrated sensitivity to change and acceptable measurement properties in previous studies, supporting its use in both clinical practice and research settings [20]. However, a rigorously translated and culturally adapted Arabic version of the SAQ has not been formally validated, despite the widespread use of Arabic across diverse populations.

Arabic is spoken in more than 20 countries, with substantial linguistic and dialectal variation. Developing an Arabic version of the SAQ that balances linguistic clarity, cultural relevance, and conceptual equivalence is therefore an essential step toward improving patient-reported assessment in Arabic-speaking SSc populations. To date, studies conducted in Arabic-speaking settings have relied largely on non-validated translations of PROMs, limiting comparability and interpretability of findings [22]. Validated translations of patient-reported outcome measures are essential for enabling meaningful comparisons of disease burden and treatment outcomes across different cultural and linguistic populations. Such instruments also facilitate the inclusion of underrepresented regions in multinational registries and collaborative clinical research in systemic sclerosis.

Therefore, the aim of the present study was to perform a cross-cultural adaptation of the Scleroderma Assessment Questionnaire into Arabic (Ar-SAQ) and to evaluate its reliability and construct validity in patients with systemic sclerosis.

## Patients and methods

### Patients and setting

A total of 193 adult patients with systemic sclerosis (SSc) fulfilling the 2013 ACR/EULAR classification criteria [23] were consecutively recruited from Rheumatology Departments and Internal Medicine Rheumatology Units across Egypt through collaboration within the Egyptian College of Rheumatology (ECR) study group. Patients with cognitive impairment that could interfere with questionnaire comprehension or interview completion were excluded. All participants provided informed consent prior to enrolment. The study protocol was approved by the Minia University, Faculty of Medicine, Institutional Review Board (MUFMIRB Approval No. 1692–6–2025). The study was conducted in accordance with the Declaration of Helsinki.

### Study design and stages

#### Translation and cross-cultural adaptation of the SAQ

The translation and cross-cultural adaptation of the Scleroderma Assessment Questionnaire (SAQ) were conducted according to internationally accepted guidelines for patient-reported outcome measures, as described by Beaton et al. [24].

- Forward translation: three independent faculty members translated the SAQ from English into Arabic (Egyptian dialect) at the same time. Two of them were rheumatologist informed about the study’s purpose and goals and the last one was a requested professional translator in the Ministry of Higher Education, Egypt, without any medical background.
- Synthesis: The translated versions were reviewed and synthesized during consensus meetings involving five rheumatologists. Linguistic clarity, conceptual equivalence, and cultural relevance were prioritized over literal translation. Classical Arabic wording was favored when possible to enhance comprehension across different Arabic-speaking regions, while maintaining intelligibility for Egyptian patients.
- Back translation: The synthesized Arabic version was back-translated into English by an independent bilingual language expert with no medical background and blinded to the original questionnaire.
- Expert Committee Review: The back-translated version was compared with the original SAQ by the expert committee, and discrepancies were discussed to ensure conceptual equivalence. A pre-final Arabic version (Ar-SAQ) was produced for cognitive testing.
- Cognitive testing (pretesting): Cognitive debriefing was conducted through face-to-face open interviews with five patients with SSc representing different educational levels. Patients were asked to comment on item clarity, relevance, and ease of understanding. Minor wording adjustments were made to improve clarity without altering item meaning. A final Arabic version and translated scoring system (Supplementary file 1) were produced after approval from the original questionnaire developer.

### Instruments and clinical assessment

All patients underwent structured interviews to collect demographic data (age, sex, education level, occupation, and residence). Clinical evaluation included disease duration, organ involvement, laboratory findings, and disease activity assessment using EUSTAR-AI [8].

### Arabic Scleroderma Assessment Questionnaire (Ar-SAQ)

The original SAQ consists of 23 items grouped into four domains: vascular (4 items), respiratory (6 items), gastrointestinal (5 items), and musculoskeletal (8 items) [25]. Items are rated on a 4-point Likert scale (0–3) assessing symptom intensity, frequency, or functional limitation.

Domain indices were calculated by dividing the sum of item scores by the number of items within each domain. The Index of Disease Status (IDS) was calculated by dividing the total SAQ score by the total number of items (23), with higher scores indicating greater patient-reported symptom burden and functional impairment. For cultural appropriateness, the musculoskeletal item referring to “holding a pack of cigarettes” was adapted to “holding a pack of tissues.” This modification preserved the intended grasping and hand function.

### Sample size calculation

The SAQ comprises 23 items across four domains. Based on psychometric validation recommendations using a minimum subject-to-item ratio of 5:1, a sample size of at least 115 participants was required. To account for potential incomplete data, the target sample size was increased by 20% to 144 participants. Between-group comparisons (limited versus diffuse SSc) were considered exploratory, and no a priori power calculation was performed for these subgroup analyses.

### Statistical analysis

All statistical analyses were performed using *IBM SPSS Statistics*, version 25 (IBM Corp., Armonk, NY, USA) and RStudio version 12.0. Quantitative data were presented by mean ± SD, while qualitative data were presented by frequency and percentage. Group comparisons between limited and diffuse SSc were conducted using the independent-samples *t*-test for continuous variables, and the χ^2^ test for categorical variables.

Internal consistency was assessed with Cronbach’s α coefficient, with values ≥ 0.70 considered acceptable, ≥ 0.80 good, and ≥ 0.90 excellent. Test–retest reliability over 4 weeks was evaluated using a two-way mixed-effects ICC (absolute agreement), interpreted as poor (< 0.50), moderate (0.50–0.75), good (0.75–0.90), or excellent (> 0.90). Construct validity was examined via Spearman correlations between SAQ domains (and total Index of Disease Status) and the EUSTAR activity index, with coefficients ≥ 0.40 indicating moderate-to-strong validity. Item–domain and item–total correlations were also analyzed by Spearman rho. Floor and ceiling effects were defined as ≥ 15% of participants scoring the minimum or maximum, respectively, and visualized with violin plots. Missing data were minimal (< 5%) and were handled using complete-case analysis. All tests were two-sided, with *p* < 0.05 considered significant.

## Results

A total of 193 SSc patients were enrolled from 15 major Egyptian universities. The figures in parentheses represent the number of participants contributed by each institution: Assiut (53), Minia (25), Kafrelsheikh (15), Zagazig (15), Beni-Suef (14), Alexandria (12), Helwan (12), Aswan (11), Cairo (10), Sohag (8), Tanta (6), Ain Shams (5), Suez Canal (4), Mansoura (2), and New Valley (1).

The mean age of participants was 44.6 ± 10.6 years, and the majority were female (171 patients, 88.6%). Sociodemographic and clinical characteristics are summarized in Table 1. The mean disease duration was 8.22 ± 5.34 years. Limited and diffuse SSc subsets were comparable with respect to age, sex distribution, education level, occupation, and disease duration.

**Table 1:** Sociodemographic and clinical characteristics of the systemic sclerosis patients (*n* = 193)

Patient characters	Total (*n* = 193)	Limited Subtype (*n* = 94)	Diffuse subtype (*n* = 99)	*P* value
**Age (years)**	44.55 ± 10.60	43.52 ± 10.24	45.53 ± 10.90	0.19
**Disease duration (years)**	8.22 ± 5.34	7.59 ± 4.96	8.81 ± 5.63	0.11
**Sex (female)**	171 (88.6%)	85 (90.4%)	86 (86.9%)	0.44
**Education**				
Not educated	45 (23.3%)	20 (21.3%)	25 (25.3%)	0.69
Low	50 (25.9%)	23 (24.5%)	27 (27.3%)	
Moderate	63 (32.6%)	31 (33%)	32 (32.3%)	
High	35 (18.1%)	20(21.3%)	15 (15.2%)	
**Occupation**				
None	23 (11.9%)	8 (8.5%)	15 (15.2%)	0.30
Housewife	115 (59.6%)	55 (58.5%)	60 (60.6%)	
Manual worker	22 (11.4%)	11 (11.7%)	11 (11.1%)	
Desk job	33 (17.1%)	20 (21.3%)	13 (13.1%)	
**Smoking status (smoker)**	19 (9.8%)	8 (8.5%)	11 (11.1%)	0.54
**Modified Rodnan skin score**	18.36 ± 9.22	12.86 ± 5.51	23.59 ± 9.0	** < 0.0001**
**Raynaud phenomenon**	193 (100%)	94 (100%)	99 (100%)	-
**Digital ulcer**	128 (66.3%)	45 (47.9%)	83 (83.3%)	** < 0.0001**
**Telangiectasia**	94 (48.7%)	54 (57.4%)	40 (40.4%)	**0.02**
**Calcinosis**	55 (28.5%)	32 (34%)	23 (23.2%)	0.10
**Muscle weakness**	66 (34.2%)	21 (22.3%)	45 (45.5%)	**0.001**
**Arthritis**	112 (58%)	51 (54.3%)	61 (61.6%)	0.30
**Gastrointestinal involvement**	168 (87%)	78 (83%)	90 (90.9%)	0.10
**Interstitial lung disease**	132 (68.4%)	43 (45.7%)	89 (89.9%)	** < 0.0001**
**Tendon friction rub**	54 (28%)	23 (24.5%)	31 (31.3%)	0.29
**CRP > 1m/dl**	133 (68.9%)	55 (58.5%)	78 (78.8%)	**0.002**
**DLCO < 70%**	102 (52.8%)	26 (27.7%)	76 (76.8%)	** < 0.0001**
**EUSTAR activity index**	5.85 ± 2.66	4.6 ± 2.44	7.03 ± 2.32	** < 0.0001**
**Active disease**	169 (87.6%)	74 (78.4%)	95 (96%)	** < 0.0001**
**Ar-SAQ Domains**				
Index of Vascular Status	1.89 ± 0.65	1.81 ± 0.62	1.96 ± 0.67	0.12
Index of Respiratory Status	1.02 ± 0.68	0.76 ± 0.65	1.26 (0.63)	** < 0.0001**
Index of Gastrointestinal Status	1.41 ± 0.65	1.26 ± 0.63	1.56 ± 0.65	**0.002**
Index of Musculoskeletal Status	1.02 ± 0.65	0.80 ± 0.59	1.2 ± 0.65	** < 0.0001**
Index of Disease Status (IDS)	1.23 ± 0.55	1.07 ± 0.52	1.43 ± 0.52	** < 0.0001**

Data are presented as frequency (percentage) or mean ± standard deviation (SD). *Ar-SAQ* scleroderma assessment questionnaire (Arabic version), *DLCO* Diffusing capacity for carbon monoxide, *CRP* C-reactive protein. *EUSTAR* European Scleroderma Trials and Research Group Activity Index. Educational levels: low equivalent to primary school, moderate equivalent to secondary school, high equivalent to college or higher educational degrees. Bold *p* value is statistically significant at < 0.05

Diffuse SSc patients demonstrated significantly higher mRSS scores (23.6 ± 9.0 vs. 12.9 ± 5.5, *p* < 0.0001), a higher frequency of digital ulcers (83.3% vs. 47.9%, *p* < 0.0001), interstitial lung disease (89.9% vs. 45.7%, *p* < 0.0001), elevated CRP (78.8% vs. 58.5%, *p* = 0.002), and reduced DLCO (76.8% vs. 27.7%, *p* < 0.0001). Diffuse SSc patients also had significantly greater EUSTAR activity scores (7.03 ± 2.32 vs. 4.60 ± 2.44, *p* < 0.0001) and a higher proportion with active disease (96% vs. 78.4%, *p* < 0.0001). All Ar-SAQ domain scores and the total Index of Disease Status were significantly higher in the diffuse SSc subset compared with the limited subset, with the exception of the vascular domain, supporting the instrument’s ability to discriminate between clinically distinct disease subsets (Table 1).

### Feasibility and score distribution

The Arabic Scleroderma Assessment Questionnaire (Ar-SAQ) was described by participating clinicians as simple, comprehensive, and easy to administer. The mean time required to complete the questionnaire was 6.53 ± 2.71 min.

**Fig. 1:**
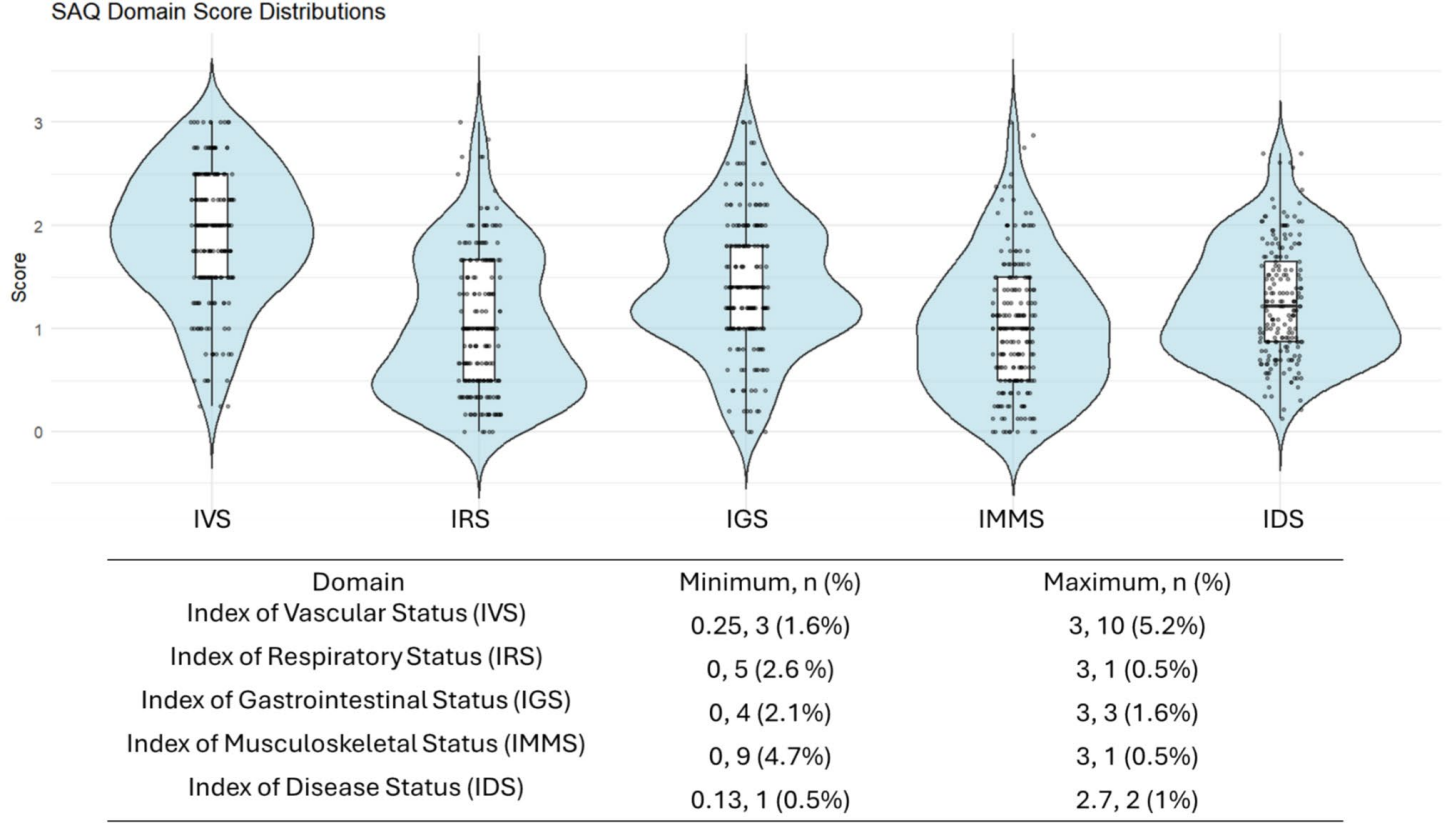
Violin plots illustrating the distribution of scores across the five Arabic SAQ domains. No floor or ceiling effects were identified, as fewer than 15% of participants achieved the minimum or maximum points

All items were understood by all participants, and no item required further clarification during administration. Score distributions across domains demonstrated adequate variability, and no floor or ceiling effects were observed, as fewer than 15% of participants achieved the minimum or maximum possible scores in any domain. Violin plots illustrating domain score distributions are presented in Fig. 1.

### Reliability

#### Test–retest reliability

Test–retest reliability was evaluated in a subgroup of 39 patients who reported no change in treatment and no clinical deterioration during the four-week retest interval. Intraclass correlation coefficients (ICC) for Ar-SAQ domains and the total score are presented in Table 2.

**Table 2:** Assessment of test-retest reliability of the SAQ domains and total score (*n* = 39)

	Test 1 mean ± SD	Test 2 mean ± SD	ICC (95% CI)	*P* value
Index of Vascular Status (IVS)	2.21 ± 0.51	2.01 ± 0.47	0.5 (0.03–0.73)	***0.02***
Index of Respiratory Status (IRS)	0.92 ± 0.61	1.05 ± 0.64	0.95 (0.90–0.97)	** < 0.0001**
Index of Gastrointestinal Status (IGS)	1.41 ± 0.48	1.43 ± 0.52	0.90 (0.81–0.95)	** < 0.0001**
Index of Musculoskeletal Status (IMMS)	0.83 ± 0.61	0.92 ± 0.60	0.92 (0.85–0.96)	** < 0.0001**
Index of Disease Status (IDS)	1.22 ± 0.47	1.25 ± 0.47	0.91 (0.83–0.95)	** < 0.0001**

Values are presented as mean (standard deviation (SD)). Bold *p* value is statistically significant at < 0.05. *ICC* Intraclass correlation coefficient, *CI* confidence interval. ICC was calculated using a two-way mixed-effects model with absolute-agreement definition

The respiratory (ICC = 0.95), gastrointestinal (ICC = 0.90), musculoskeletal (ICC = 0.92), and total Index of Disease Status (ICC = 0.91) domains demonstrated good to excellent reliability, indicating strong temporal stability. The vascular domain showed moderate reliability (ICC = 0.50, 95% CI 0.03–0.73).

The wide confidence interval observed for the vascular domain indicates greater measurement uncertainty and should be interpreted with caution, likely reflecting the known short-term variability of vascular manifestations in systemic sclerosis.

The mean differences between test and retest scores were small, confirming consistent performance of the translated instrument across administrations.

### Internal consistency

Internal consistency analyses demonstrated acceptable to excellent reliability across all Ar-SAQ domains (Table 3). Cronbach’s alpha coefficients were 0.787 for the vascular domain, 0.893 for the respiratory domain, 0.800 for the gastrointestinal domain, and 0.918 for the musculoskeletal domain. The overall Index of Disease Status demonstrated excellent internal consistency (Cronbach’s alpha = 0.939).

**Table 3:** Assessment of internal consistency of SAQ (*n* = 193)

Ar-SAQ categories	Cronbach’s Alpha	*N* of Items
Index of Vascular Status (IVS)	0.787	4
Index of Respiratory Status (IRS)	0.893	6
Index of Gastrointestinal Status (IGS)	0.800	5
Index of Musculoskeletal Status (IMMS)	0.918	8
Index of Disease Status (IDS)	0.939	23

*Ar-SAQ* Scleroderma Assessment Questionnaire (Arabic version), Internal consistency reliability was evaluated using Cronbach’s alpha coefficient

A Cronbach’s alpha ≥ 0.70 indicated acceptable; α ≥ 0.80 good; α ≥ 0.90 excellent

These findings indicate strong homogeneity among items within each domain and support the internal reliability of the Arabic version of the SAQ.

### Construct validity

Construct validity was assessed by examining correlations between Ar-SAQ domain and total scores and the European Scleroderma Trials and Research Group activity index (EUSTAR-AI) (Table 4).

**Table 4:** Convergent Validity (Spearman Correlations with EUSTAR Activity Index)

Ar-SAQ categories	Convergent Validity (EUSTAR)	
	Spearman *r*	*P* value
Index of Vascular Status (IVS)	0.354	** < 0.0001**
Index of Respiratory Status (IRS)	0.408	** < 0.0001**
Index of Gastrointestinal Status (IGS)	0.314	** < 0.0001**
Index of Musculoskeletal Status (IMMS)	0.394	** < 0.0001**
Index of Disease Status (IDS)	0.451	** < 0.0001**

*Ar-SAQ* Scleroderma Assessment Questionnaire (Arabic version), *EUSTAR* European Scleroderma Trials and Research Group Activity Index

Convergent validity was assessed using Spearman rank correlation analysis

Bold *p* value is statistically significant at < 0.05

All Ar-SAQ domains demonstrated statistically significant positive correlations with the EUSTAR activity index, with correlation coefficients ranging from 0.31 to 0.45 (all *p* < 0.0001). The strongest correlation was observed for the total Index of Disease Status (*r* = 0.451).

These results indicate moderate construct validity and support the ability of the Ar-SAQ to reflect patient-reported disease status associated with clinician-assessed disease activity.

### Item–total and item–domain correlations

Item-level analyses demonstrated strong correlations between individual items and their respective domain scores, with Spearman correlation coefficients ranging from 0.66 to 0.87. Correlations between individual items and the total Index of Disease Status ranged from 0.49 to 0.74 (all *p* < 0.0001).

Domain–total correlations were also high for all domains (IVS = 0.719; IRS = 0.860; IGS = 0.803; IMMS = 0.890), confirming robust internal construct validity across all Ar-SAQ subscales (Table 5).

**Table 5:** Item–Total and Item–Domain Correlations of the Arabic SAQ (*n* = 193)

	Item–domain r	Item–IDS r
Vascular Question 1	0.743**	0.504**
Vascular Question 2	0.783**	0.534**
Vascular Question 3	0.824**	0.562**
Vascular Question 4	0.782**	0.648**
Index of Vascular Status (IVS)		0.719**
Respiratory Question 1	0.805**	0.720**
Respiratory Question 2	0.871**	0.726**
Respiratory Question 3	0.855**	0.735**
Respiratory Question 4	0.804**	0.691**
Respiratory Question 5	0.768**	0.677**
Respiratory Question 6	0.747**	0.618**
Index of Respiratory Status (IRS)		0.860**
Gastrointestinal Question 1	0.835**	0.687**
Gastrointestinal Question 2	0.663**	0.583**
Gastrointestinal Question 3	0.714**	0.495**
Gastrointestinal Question 4	0.795**	0.672**
Gastrointestinal Question 5	0.720**	0.562**
Index of Gastrointestinal Status (IGS)		0.803**
Musculoskeletal Question 1	0.717**	0.690**
Musculoskeletal Question 2	0.715**	0.744**
Musculoskeletal Question 3	0.815**	0.713**
Musculoskeletal Question 4	0.850**	0.733**
Musculoskeletal Question 5	0.828**	0.682**
Musculoskeletal Question 6	0.795**	0.682**
Musculoskeletal Question 7	0.828**	0.704**
Musculoskeletal Question 8	0.834**	0.724**
Index of Musculoskeletal Status (IMMS)		0.890**

***r***, Spearman correlation coefficient. ***Item-Domain r***, correlation between each item and its corresponding domain total score. ***Item-IDS r***, correlation between each item and the total SAQ score (Index of Disease Status, IDS). ** = *p* < 0.0001 (statistically significant). Correlations < 0.30 indicate acceptable association; higher values reflect stronger internal construct validity

## Discussion

This study presents the first cross-culturally adapted and psychometrically evaluated Arabic version of the Scleroderma Assessment Questionnaire for use in Arabic-speaking systemic sclerosis (SSc) populations. Given the urgent need for reliable patient-reported outcome measures (PROMs) in multisystem rheumatologic diseases, the Ar-SAQ provides a culturally adapted tool capable of capturing subjective symptoms that may reflect reversible disease processes [19, 20]. Early diagnosis of SSc is challenging due to the absence of characteristic early symptoms, and diagnostic delays may permit irreversible progression of organ involvement. Identifying early indicators of skin and internal organ disease is therefore crucial to prevent further damage [26]. Major alteration of the natural history of SSc is limited with current treatments, and the development of novel therapies has been hampered, in part, by the lack of fully validated multi-system outcome measures [16].

Differentiating activity from chronicity or damage is one of the key aspects in treatment of all rheumatological disorders and dictates the aggressiveness of treatment strategies employed. This differentiation is especially difficult in SSc, owing to the incomplete understanding of the multifaceted pathogenesis of this complex disorder. Clinical features traditionally thought to represent irreversible damage may, in fact, be reversible disease activity [27]. Disease status must therefore be assessed through a combination of activity, severity, and damage indices. Disease activity refers to the dynamic, potentially reversible component of disease that can improve spontaneously or with therapy [15].

To date, no universally available gold standard method for assessing overall SSc activity [17]. Instruments such as the EscSG Activity Index and the updated r-EUSTAR Activity Index can quantify activity and predict major organ involvement in clinical practice. The Composite Response Index in SSc (CRISS) [28] is particularly useful in early diffuse SSc in clinical trials [17]. Interestingly, on considering the disease activity score 28 using ESR (DAS28-ESR) and CRP (DAS28-CRP), the Simplified Disease Activity Index (SDAI) and the Clinical Disease Activity Index (CDAI) used in RA for SSc patients, all four disease activity composite indices were valid measures for assessing arthritis in SSc; the DAS28-ESR showed the best performance regarding reliability and construct validity [29]. Relationships among activity, damage, and severity vary widely between cohorts, driven by population characteristics [30].

Disease activity in SSc declines over time, and patients with diffuse disease consistently exhibit higher activity scores. Depression has been shown to influence patient-reported activity scores, reflecting the subjective components of these instruments and highlighting the need to consider psychological factors in outcome assessment [31].

Originally Serbian and translated to English, the SAQ is a sensitive measurement to demonstrate change in patients with SSc over time [20]. The present adaptation followed all recommended steps by Beaton et al., involving five medical translation experts and a bilingual English–Arabic language specialist. Recent advances in artificial intelligence have enabled automated translation tools that can assist in preliminary linguistic translation of questionnaires. However, current methodological guidelines for cross-cultural adaptation of patient-reported outcome measures emphasize that expert forward and backward translation, multidisciplinary review, and cognitive testing with patients remain essential steps to ensure conceptual equivalence and cultural relevance [24]. AI-assisted translation may therefore support the adaptation process but cannot replace rigorous expert-driven validation. The final Arabic version (Ar-SAQ) was easily understood across multiple regions in Egypt despite dialectal variability. Pre-testing confirmed clarity and feasibility, with a mean administration time of 6.53 ± 2.71 min.

The development, translation, and cross-cultural adaptation process must be precise and rigorous as it is not exactly the original score, making an optimal international comparison defective. To evaluate the reliability of the Ar-SAQ, test–retest correlations and agreement statistics were examined. Although individual item reliability varied reflecting the inherently subjective nature of symptom reporting, the overall reproducibility of the instrument was satisfactory. Similar findings were reported in the original SAQ validation [21].

The Ar-SAQ was simple to apply and score, and its use has begun to appear in local clinical research, although previous applications relied on non-validated translations [22]. Wider implementation may benefit from larger and longitudinal studies and from inclusion of early and incomplete SSc subsets to capture the full disease spectrum. Given the known geographical and ethnic variability in SSc manifestations even within the same country, further evaluation of the SAQ in different Arab populations is recommended to support universal clinical use. The availability of a validated Arabic version of the SAQ may also facilitate the inclusion of Arabic-speaking patients in multinational registries and collaborative studies of systemic sclerosis. Standardized patient-reported outcome measures enable harmonized data collection across cohorts and improve the comparability of clinical research conducted in different geographic regions.

The Ar-SAQ demonstrated overall satisfactory test–retest reliability. ICC values ranged from 0.50 to 0.95, indicating moderate to excellent reproducibility over a four-week interval. The respiratory, gastrointestinal, musculoskeletal, and total IDS domains exhibited good to excellent reliability (ICC = 0.90–0.95), confirming strong temporal stability suitable for clinical and research applications.

The vascular domain (IVS) showed only moderate reliability (ICC = 0.50). This finding is consistent with the biological behaviour of vascular manifestations in SSc, which fluctuate markedly due to environmental changes, stress, and variable microvascular reactivity [32].

Several limitations should be acknowledged. First, factorial validity and responsiveness to change were not assessed, and therefore the present findings should be interpreted as preliminary with respect to the full psychometric profile of the instrument. Second, test–retest reliability was evaluated in a relatively small subgroup, although this sample size is consistent with PROM validation standards [25]. Third, the study population was limited to Egyptian patients; further validation in other Arabic-speaking populations is warranted to support broader regional generalizability.

## Conclusion

The cross-cultural adaptation of the SAQ questionnaire for use in Egypt was effective. The developed Arabic version of the SAQ is a reliable and valid instrument for measuring the current disease activity in SSc patients, especially for the domains on vascular, respiratory, gastrointestinal and musculoskeletal domains and items. Its availability may also facilitate the inclusion of Arabic-speaking populations in multinational studies and registries of systemic sclerosis.

## Supplementary Information

Below is the link to the electronic supplementary material.

ESM 1 (DOCX 19.5 KB)

## Data Availability

The data that support the findings of this study are available from the corresponding author upon reasonable request.
