# Supplementary material for: Cross-cultural adaptation, reliability and construct validity of the Arabic Scleroderma Assessment Questionnaire in Egyptian patients with systemic sclerosis
Source: Clin Rheumatol. 2026 Apr 9;45(6):3405–14. doi: 10.1007/s10067-026-08055-5 (PMC13249707; doi:10.1007/s10067-026-08055-5)
Supplement: Supplementary file 1 — (DOCX 19.5 KB) [file 10067_2026_8055_MOESM1_ESM.docx]

| **Vascular dysfunction** | Do you feel pain in fingers when they are exposed to a cold environment? | هل تشعر بألم في الأصابع عندما تتعرض للبرودة؟ | **اختلال الأوعية الدموية** | **B** |
| --- | --- | --- | --- | --- |
|  | Do your fingers change color (turn white or blue) when they are exposed to a cold environment? | هل يتغير لون أصابعك (تصبح بيضاء او زرقاء اللون) عندما تتعرض للبرودة؟ |  | **A** |
|  | Do you feel pain in chest when the weather is cold? | هل تشعر بألم في الصدر عندما يصبح الجو بارد؟ |  | **A** |
|  | Do you feel pain in your fingers when touching or holding objects? | هل تشعر بألم في أصابعك عند لمس أو إمساك الأشياء؟ |  | **A** |
| **Respiratory dysfunction** | Do you feel you are short of breath while climbing twenty stairs? | هل تشعر بضيق في التنفس (نهجان) عند صعود عشرين درجة من السلم (دور/طابق واحد)؟ | **اختلال الجهاز التنفسي** | **A** |
|  | Do you feel you are short of breath when walking on flat ground? | هل تشعر بضيق في التنفس (نهجان) عند المشي على أرض مستوية؟ |  | **A** |
|  | Do you feel you are short of breath when dressing? | هل تشعر بضيق في التنفس (نهجان) أثناء ارتداء الملابس؟ |  | **A** |
|  | Do you feel you are short of breath when sitting? | هل تشعر بضيق في التنفس (نهجان) أثناء الجلوس؟ |  | **A** |
|  | Do you cough? | هل تشتكى من السعال/الكحة؟ |  | **B** |
|  | Do you cough up phlegm? | هل يصاحب السعال/الكحة بلغم (بصاق) ؟ |  | **B** |
| **Gastrointestinal dysfunction** | Does a bite of a firm food (for example bread) lie behind your breastbone when you swallow? | هل تشعر بصعوبة في بلع لقمة من الطعام الصلب مثل قطعة من الخبز، فتشعر بأنها تعلق في حلقك؟ | **اختلال الجهاز الهضمي** | **B** |
|  | Does a sip of liquid (for example water or tea) lie behind your breastbone when you swallow? | هل تشعر بصعوبة في بلع السوائل مثل (رشفة ماء أو شاي)، فتشعر بأنها تعلق في حلقك؟ |  | **B** |
|  | Do you have heartburn? | هل تعاني من حرقة في المعدة؟ |  | **B** |
|  | Do you feel pain when swallowing? | هل تشعر بألم أثناء البلع؟ |  | **A** |
|  | Do you have constipation or diarrhea? | هل تعاني من إمساك أو إسهال؟ |  | **B** |
| **Musculoskeletal dysfunction** | Do you drop objects frequently when holding them (for example a bar of soap, a glass or a pack of cigarettes)? | هل يتكرر سقوط الأدوات منك عندما تمسكها (مثل الصابون أو الكوب أو علبة مناديل ورقية)؟ | **اختلال الجهاز العضلي الهيكلي** | **B** |
|  | Are you able to open your mouth wide enough to bite an apple? | هل بإمكانك فتح فمك بما يكفى لتقطم تفاحة؟ |  | **C** |
|  | Are you able to hold a pen and write? | هل تستطيع الإمساك بقلم والكتابة به؟ |  | **C** |
|  | Are you able to button and unbutton your shirt? | هل تستطيع غلق وفتح أزرار قميصك؟ |  | **C** |
|  | Are you able to hold a knife and cut bread? | هل تستطيع الإمساك بسكين وتقطيع الخبز؟ |  | **C** |
|  | Are you able to get out of bed without someone’s help? | هل أنت قادر على النهوض من السرير بدون مساعدة من آخرين؟ |  | **C** |
|  | Are you able to dry your entire body with a towel? | هل تستطيع تجفيف جسمك بالكامل بالمنشفة (فوطة)؟ |  | **C** |
|  | Are you able to make your bed? | هل تستطيع ترتيب سريرك؟ |  | **C** |

Answering categories: A intensity of symptoms (no=0, some=1, moderate=2, very intensive=3), B frequency of symptoms (never=0, sometimes=1, frequently=2, always=3), C ability to perform activities (without difficulty=0, with some difficulty=1, with much difficulty=2, no able to do=3)

A **شدة الأعراض** (لا توجد الأعراض=0، بشكل بسيط=1، متوسط الشدة=2، شديد جدًا=3)،

B **تكرار الأعراض** (لم تتكرر الأعراض أبدًا=0، تتكرر الأعراض على فترات متباعدة =1، تتكرر الأعراض في أغلب الفترات=2، تتكرر الأعراض دائمًا=3)

C **القدرة على أداء الأنشطة** (بدون صعوبة=0، مع بعض الصعوبة=1، مع صعوبة شديدة=2، غير قادر على الأداء=3).
